# Predicting unplanned medical visits among patients with diabetes: translation from machine learning to clinical implementation

**DOI:** 10.1186/s12911-021-01474-1

**Published:** 2021-03-31

**Authors:** Arielle Selya, Drake Anshutz, Emily Griese, Tess L. Weber, Benson Hsu, Cheryl Ward

**Affiliations:** 1 Department of Population Health, University of North Dakota School of Medicine and Health Sciences, Grand Forks, ND USA; 2 Behavioral Sciences Group, Sanford Research, 2301 East 60th Street North, Sioux Falls, SD 57104 USA; 3 Department of Pediatrics, University of South Dakota Sanford School of Medicine, Sioux Falls, SD USA; 4 Pinney Associates, Inc., Pittsburgh, PA USA; 5 Advanced Analytics, St. Luke’s Health System, Boise, ID USA; 6 Sanford Heath Plan, Sioux Falls, SD USA; 7 EDCO Health Information Systems, Sioux Falls, SD USA

**Keywords:** Diabetes, Unplanned medical visits, Machine learning, Predictive model

## Abstract

**Background:**

Diabetes is a medical and economic burden in the United States. In this study, a machine learning predictive model was developed to predict unplanned medical visits among patients with diabetes, and findings were used to design a clinical intervention in the sponsoring healthcare organization. This study presents a case study of how predictive analytics can inform clinical actions, and describes practical factors that must be incorporated in order to translate research into clinical practice.

**Methods:**

Data were drawn from electronic medical records (EMRs) from a large healthcare organization in the Northern Plains region of the US, from adult (≥ 18 years old) patients with type 1 or type 2 diabetes who received care at least once during the 3-year period. A variety of machine-learning classification models were run using standard EMR variables as predictors (age, body mass index (BMI), systolic blood pressure (BP), diastolic BP, low-density lipoprotein, high-density lipoprotein (HDL), glycohemoglobin (A1C), smoking status, number of diagnoses and number of prescriptions). The best-performing model after cross-validation testing was analyzed to identify strongest predictors.

**Results:**

The best-performing model was a linear-basis support vector machine, which achieved a balanced accuracy (average of sensitivity and specificity) of 65.7%. This model outperformed a conventional logistic regression by 0.4 percentage points. A sensitivity analysis identified BP and HDL as the strongest predictors, such that disrupting these variables with random noise decreased the model’s overall balanced accuracy by 1.3 and 1.4 percentage points, respectively. These recommendations, along with stakeholder engagement, behavioral economics strategies, and implementation science principles helped to inform the design of a clinical intervention targeting behavioral changes.

**Conclusion:**

Our machine-learning predictive model more accurately predicted unplanned medical visits among patients with diabetes, relative to conventional models. Post-hoc analysis of the model was used for hypothesis generation, namely that HDL and BP are the strongest contributors to unplanned medical visits among patients with diabetes. These findings were translated into a clinical intervention now being piloted at the sponsoring healthcare organization. In this way, this predictive model can be used in moving from prediction to implementation and improved diabetes care management in clinical settings.

## Background

There are approximately 1.5 million new diabetes diagnoses among people 18 years and over every year, and in 2018, approximately 34.2 million persons (10.5%) in the US had diabetes [1]. In 2017, 83,564 deaths were attributed to diabetes in the United States, and that year, diabetes was the 7th leading cause of death in the United States (25.7 deaths per 100,000 population) [1].

Diabetes imposes significant healthcare utilization and costs [2]. Americans with diabetes in 2017 spent approximately $16,700 annually in health care costs, 2.3 times higher than those without diabetes [3]. Total costs of diabetes in 2017 were $327 billion annually, of which $237 billion were in direct medical costs [3]. In addition, there is a positive relationship between lack of health insurance and prevalence of diagnosed diabetes, exacerbating the risks for uninsured Americans [4]. By 2034, the population with diabetes is expected to increase by 100% and the cost is expected to increase by 53% [5].

Patients with diabetes generally have increased healthcare utilization, including planned visits (e.g. clinic visits, outpatient departments), as well as unplanned visits (e.g. emergency department and urgent care visits), compared to those without diabetes [3, 6]. The 2011 National Health Interview Survey Diabetes revealed that 30% of diabetic patients had at least one emergency department visit within the last year, compared to only 20% of the general population [6]. The majority of emergency department visits among patients with diabetes are likely related to acute glycemic complications (hyperglycemia and hypoglycemia) [6]; however, most adults with diabetes have at least one comorbid chronic condition [7] which could contribute to these visits as well. Unplanned visits typically present a greater burden to patients and insurers due to the higher cost of these visits.

Additionally, social and behavioral factors are associated with unplanned medical visits among the population of patients with diabetes. Lower socioeconomic status, longer disease duration, disease severity, and co-morbid depression are all significant determinants of unplanned medical visits and hospitalizations [8]. More precisely, patients with diabetes with very high current depressive symptoms were two times more likely to have an unplanned emergency department visit, and patients who were diagnosed more than 10 years ago were 1.3 times as likely to have an unplanned emergency department visit [8]. Additionally, cigarette smoking is associated with a greater likelihood of unplanned medical visits [9]. However, unplanned visits remain a high-impact problem for patients and healthcare systems alike, highlighting the need for improved prediction models that can be implemented clinically.

Because of the increased risks and associated costs for patients with diabetes, there is a significant need to improve prediction capabilities aimed at reducing unplanned medical visits for this group of patients. A majority of medical risk prediction models have been developed using stepwise logistic regression, while machine learning classification methods have been largely unexplored [10]. Machine learning methods offer the additional possibility to improve prediction based on pattern detection of many variables simultaneously, as has been shown in applications on predicting obesity [11] and compliance with dietary recommendations [12], predicting metabolic syndrome from physical characteristics and lab results [13], identifying binge drinkers from parenting variables [14] and drinking motives [15], and predicting high blood pressure using body measures [16]. The current study utilizes electronic medical record (EMR) data from a large healthcare system and develops a machine learning based predictive model to predict any versus no unplanned medical visits over a 3-year period among adult patients with diabetes. We also discuss how the findings of this predictive model were translated into a clinical intervention currently underway at the sponsoring healthcare organization.

## Methods

### Sample

Data were obtained from electronic medical records (EMRs) in EPIC from Sanford Health, a not-for-profit rural healthcare system that primarily serves South Dakota, North Dakota, Northern and Southwest Minnesota, Northwest Iowa, and parts of Nebraska. Sanford Health includes roughly 44 hospitals, 1382 physicians and 9703 nurses delivering care in more than 80 specialty areas. All data were de-identified according to the Health Insurance Portability and Accountability Act HIPAA de-identification method Safe Harbor § 164.514(b)(2). The dataset included records from all patients who visited a Sanford healthcare facility between January 1, 2014 and December 30, 2016 (*N* = 1,143,028). Only adult patients (age ≥ 18; *N* = 875,168) with a diagnosis of diabetes (ICD-10 codes E10.xx and E11.xx; *N* = 67,575) were included in the current study. Further, only patients who reported a residential zip code in Minnesota (MN), North Dakota (ND), or South Dakota (SD) were included in the current study (*N* = 63,781), due to low sample sizes in other states. Finally, patients who had missing data on the outcome variable of unplanned medical visits or any of the predictor variables were excluded, for a final sample size of *N* = 43,831.

### Measures

The outcome was any versus no unplanned medical visits during the 3-year period over which EMR data were collected. This outcome was derived from four separate variables: emergency department visits, hospitalizations, hospital observations, and urgent care visits. All four types of visits were summed and dichotomized as ≥ 1 versus 0 unplanned medical visits.

Predictor variables included all numeric variables that were common and readily available in Sanford’s EMRs. Ten variables were selected and are described in detail below.

Age was measured in years at time of initial analyses (12/1/2016).

Body mass index (BMI) was obtained from EMRs as kg/m^2^. Extreme values (< 15 or > 60) were assumed to be errors and were set to missing. Values from the most recent visit in the 3-year period were used, as this was the only measure in the dataset provided by the sponsoring healthcare organization (see Limitations).

Blood pressure (BP) was obtained in mm/Hg. Values from the most recent visit in the 3-year period were used, as this was the only measure in the dataset provided by the sponsoring healthcare organization (see Limitations). Systolic BP and diastolic BP were included as two separate variables.

Serum cholesterol was obtained as both low-density lipoprotein (LDL) and high-density lipoprotein (HDL) in mg/dL. Extreme values in HDL (< 10 or > 100) or LDL (< 20 or > 200) were assumed to be errors and were set to missing. Values from the most recent laboratory result were used, as this was the only measure in the dataset provided by the sponsoring healthcare organization (see Limitations). LDL and HDL were analyzed as two separate variables.

Glycohemoglobin (A1C) was measured from the most recent laboratory result, as this was the only measure in the dataset provided by the sponsoring healthcare organization (see Limitations). A1C values below 4 or above 15 were assumed to be errors and were set to missing.

Ranked smoking status was obtained by patient self-report as a vital sign on their most recent visit, as this was the only measure in the dataset provided by the sponsoring healthcare organization (see Limitations). A ranked variable was created as follows from the several possible response categories, with higher values indicating more smoke exposure: never smoker (0), passive smoker (1), former smoker (2), current some day smoker (3), current everyday smoker, light tobacco smoker, or heavy tobacco smoker (4).

Number of diagnoses on “problem list” was derived from the most recently available list over the 3-year period.

Number of prescriptions were aggregated over the 3-year period and was used as a numeric variable.

### Analyses

#### Machine learning

All analyses predicted the unplanned medical visit status of each patient (i.e., which patients had at least one versus no unplanned medical visits in the 3-year period), and this classification task was based on the 10 EMR variables above (age, BMI, systolic and diastolic BP, HDL and LDL cholesterol, A1C, ranked smoking status, number of diagnoses on the patient’s “problem list,” and the number of prescriptions in the 3-year period). Four types of machine learning were utilized: discriminant analysis (linear and quadratic), support vector machines (SVM; linear basis and radial basis), single-layer artificial neural nets (NN’s) triple-layer deep nets (DNN’s), and extreme gradient boosting (XG boost). R software [17] was used for all analyses, including the packages MASS for discriminant analysis [18], e1071 for SVM’s [19], nnet for single-layer NN’s [18], deepnet for triple-layer DNN’s [20], and xgboost for XG boost [21]. A logistic regression was run for purposes of comparing machine learning results with conventional prediction approaches. All R code for this project is publicly available on github at: https://github.com/ArielleSelya/Diabetes-Predictive-Model.

#### Cross-validation testing

Since classifiers are susceptible to overtraining (i.e. when the classifier can predict the training dataset with high accuracy, but fits noise and thus has not learned patterns that generalize to other datasets), cross-validation testing is important to identify models that have detected patterns that are truly important in the prediction task. Cross-validation testing is performed by partitioning all available data points into a training set and a testing set; the classifier is trained on the data from the training set, and the generalization of the prediction task learned by the classifier is tested using the data from the testing set. Nested cross-validation is a procedure which further reduces overfitting by performing this cross-validation procedure on a subset of the data (“inner fold,” which is then split into training and test sets), selecting the optimal parameters, and testing on the remaining data in the “outer fold.” This procedure is important in selecting optimal hyperparameters for the model, as using the same dataset for generalization as well as model selection introduces bias [22].

In particular for this study, five-fold cross-validation was used. For models with hyperparameters to be optimized (i.e. SVM, NN, DNN, XG boost), nested cross-validation was used to select optimize hyperparameters. Five-fold cross-validation was used on the outer loop. Inside each outer loop, the possible hyperparameters were looped over, with balanced accuracy for each hyperparameter being evaluated by another “inner” loop of five-fold cross-validation. The hyperparameters and balanced accuracy for each outer fold were recorded, and the most common set of hyperparameters were selected as the “final” model.

Both training performance (prediction on the training dataset) and generalization performance (prediction on the testing set) were assessed using confusion matrices. SVM, NN, DNN, and XG boost classifiers were optimized by running several iterations over different parameter values. For SVM, possible cost parameters were 0.1, 0.5, 1, 5, 10, 25, and 50; and for radial SVM, possible gamma parameters were 0.0001, 0.001, 0.003, 0.007, 0.1, 0.5, and 1. For single-layer NN’s, possible hidden layer sizes were 1, 2, 5, 10, 15, and 20; the possible maximum training iterations were 100, 150, and 200; and the possible decay parameters were 0, 0.1, 0.3, 0.5, and 0.9. For triple-layer NN’s, possible sizes of the first hidden layer were 1, 5, 10, 15, and 20; for the second and third hidden layers, possible values were 1, 5, 10, and 20; possible learning rates were 0, 0.1, 0.5, and 1; possible momentum values of the learning rate were 0, 0.1, 0.5, and 1; and possible numbers of training iterations were 10 and 20. For XG boost, possible maximum depth values were 3, 6, 10, 15, and 20; possible eta values (learning rate) were 0.01, 0.5, 0.1, 0.3, and 0.6; possible values for number of rounds were 50, 100, 150, and 200; possible gamma values were 0, 0.5, 1, 5, 10, and 25; and possible ratios of columns variables per tree were 0.1, 0.5, and 1.0. For each classifier, the model with the highest performance (see next section) is reported.

#### Performance metric

Many performance metrics exist for classifiers, and we selected one for the current application for the following reasons. First, class imbalance in a dataset (here, 57% of the sample with unplanned medical visits vs. 43% without) can impact classifier performance, such that the classifier may show bias towards the more common class. Since the conventional definition of overall model accuracy ($\frac{1 - incorrect\;predictions}{{total\;predictions}}$) is sensitive to class imbalance, the imbalance would need to be taken into account if using this traditional accuracy metric. However, using this accuracy metric tends to result in overprediction of positives, often at the expense of a high false-alarm rate; this is undesirable in clinical settings due to the cost and potential harm resulting from false alarms (i.e. providing interventions or treatment to patients who do not need it). For example, strict criteria for prostate cancer screening have historically erred on the side of identifying positives (both true and false), resulting in high rates of unnecessary biopsies and other treatments for patients who (as is now understood) were unlikely to ever show clinical symptoms of prostate cancer. Thus, given the clinical applications of the current study, it is essential to maximize both the true positive (sensitivity) and true negative (specificity) rates. Previous work has shown that maximizing the sum of sensitivity and specificity is appropriate for clinical applications with trade-offs between accurate risk detection and minimizing false alarms [23]. Here, we use such a measure: $\frac{sensitivity + specificity}{2}$ where we divide by 2 in order for more intuitive interpretation, i.e. to average across the correct predictions within each class. This metric, “balanced accuracy,” has the advantage of selecting a model based on the maximum true positive and true negative rates (i.e. minimizing both false positives and false negatives), which is appropriate for clinical applications [23].

Since balanced accuracy contains two possible categories and averages sensitivity and specificity, chance performance is 50%. Notably, this cutoff holds even in cases of imbalance (here, the 57% default sensitivity would be balanced by the 43% specificity, resulting in a performance of 50%). Classifier performance versus chance was measured using a binomial test of the success rate out of the 1000 cross-validation iterations.

#### Sensitivity testing

In order to derive clinical implications from the predictive model, it is valuable to know which variables are most strongly predictive of unplanned medical visits. Although being important for prediction does not necessarily indicate causality, many of the modifiable predictors (A1C, BMI, BP, cholesterol, smoking) do have plausible causal effects on diabetes and its complications. Thus, in order to determine the modifiable variables that are most strongly indicative of unplanned medical visits, a variant of sensitivity testing was performed: for one variable at a time, random noise was added to that variable. These random values were drawn from a normal distribution with the same mean as the variable being tested, and a standard deviation of 30% that of that variable. For the sensitivity testing, cross-validation was done using 25 iterations of hold-20%-out repeated subsampling; this cross-validation procedure was different from the approach used in the main analysis due to the greater need for precision introduced by adding noise. These were compared to the base-case balanced accuracy using the original dataset and the classifier with the optimal hyperparameters obtained above (which was re-run using this cross-validation method, for comparability). Larger disruptions to the balanced generalization accuracy as a result of disrupting the information content of that variable (i.e. by adding noise) indicates a greater importance of that variable to the prediction task, and potentially as a clinical target for intervention.

#### Clinical intervention development

The process by which the above findings were incorporated into a clinical intervention are discussed, along with other factors including patient and physician engagement, interfacing with clinical operations, and decision-making under real-world practical limitations.

## Results

Table 1 shows the characteristics of the sample, summarized by patients who did versus did not have unplanned medical visits during the 3-year period. Patients with at least one unplanned visit tended to be slightly older (66 vs. 65 years old), rank higher on the smoking scale (2 vs. 1), have more diagnoses on the problem list (4 vs. 3), have lower HDL values (42 vs. 44), and have been prescribed considerably more medications over the 3-year period (205 vs. 88) (all *p* < 0.05). The two groups had similar mean levels of diastolic blood pressure, but those with at least one unplanned visit had a wider interquartile range (IQR: 64–80 vs. 66–80), resulting in a statistically significant difference. Similarly, the two groups had similar mean levels of A1C, but those with at least one unplanned visit had a wider IQR (6.3–7.9 vs. 6.3–7.8), resulting in a significantly different difference. Though these differences are minor, they are statistically significant in part because of the large sample size. The significance should be interpreted along with the effect size; the differences reported here are unlikely to be clinically meaningful. No significant difference was observed for BMI, systolic blood pressure or LDL cholesterol (*p* > 0.05).

**Table 1:** Characteristics of patients with diabetes by unplanned visit status

Predictor variable	No unplanned visits (*N* = 18,771)	≥ 1 Unplanned visits (*N* = 25,060)	*p* value
Age	**65 (55–74)**	**66 (55–76)**	**< .0001**
BMI	32.3 (28.3–37.0)	32.2 (28.0–37.3)	= .2454
Systolic BP	126.0 (118.0–134.0)	126.0 (116.0–136)	= .0089
Diastolic BP	**72.0 (66.0–80.0)**	**72.0 (64.0–80.0)**	**< .0001**
LDL cholesterol	85.0 (67.0–106.0)	84.0 (65.0–106.0)	= .0053
HDL cholesterol	**44.0 (37.0–53.0)**	**42.0 (35.0–52.0)**	**< .0001**
A1C	**6.9 (6.3–7.8)**	**6.9 (6.3–7.9)**	**= .0001**
Ranked smoking status	**1.0 (0.0–2.0)**	**2.0 (0.0–2.0)**	**< .0001**
Number of diagnoses on problem list	**3.0 (2.0–4.0)**	**4.0 (3.0–6.0)**	**< .0001**
Number of prescriptions	**88.0 (40.0–179.0)**	**205.0 (96.0–408.0)**	**< .0001**

Variables are summarized as median (interquartile range). *A1C* glycohemoglobin. *BMI* body mass index. *BP* blood pressure. *HDL* high-density lipoprotein. *LDL* low-density lipoprotein. *p* values are based on t-tests of each variable across groups (any vs. no unplanned visits). Bold: *p* < .05

Table 2 shows the balanced accuracy of each type of classifier after optimization (i.e. using optimal parameter settings), averaged across the 1000 cross-validation runs. Logistic regression (bottom row) is intended as a comparison, as it only models main effects of each predictor and does not contain any interaction terms. Logistic regression performed reasonably well, with a sensitivity (true positive rate) of 70.2% and a specificity (true negative rate) of 60.4%. XG boost classifiers found the highest sensitivity of all models (83.3%), but specificity was low (33.9%); thus, this was considered a low performing model, especially relative to logistic regression, due to its inability to distinguish between classes. For similar reasons, linear discriminant analysis also resulted in a low performing model, with high sensitivity (75.2%) and low specificity (50.8%). Linear SVM was the only model that outperformed the logistic regression, at 65.7% balanced accuracy, with the sensitivity (60.2%) and specificity (71.1%) both being significantly above chance, and significantly higher than logistic regression (*p* = 0.03 according to a t-test of the balanced accuracy). Single and triple hidden layer neural networks (NN’s and DNN’s, respectively) were found to underperform in comparison to logistic regression balanced accuracy, and were also highly variable across cross-validation folds, as were XG boost classifiers. Table 3 shows the best-performing hyper-parameters within each outer fold.

**Table 2:** Generalization performance of classifiers with optimized parameters, presented as confusion matrices and balanced accuracy ± standard deviation across five-fold cross-validation

Classifier	Most stable parameters across outer folds		Predicted: no unplanned visits	Predicted: ≥ 1 unplanned visit	
Linear discriminant analysis	N/A	Actual: No Unplanned Visits	50.8% ± 1.4%	49.2% ± 1.4%	
		Actual: ≥ 1 Unplanned Visit	24.8% ± 1.0%	75.2% ± 1.0%	
		Average	63.0% ± 0.7%		
Quadratic discriminant analysis	N/A	Actual: No Unplanned Visits	82.5% ± 0.6%	17.5% ± 0.6%	
		Actual: ≥ 1 Unplanned Visit	56.3% ± 0.8%	43.7% ± 0.8%	
		Average	63.3% ± 0.4%		
Linear SVM	Cost = 25	Actual: No Unplanned Visits	71.1% ± 0.8%	28.9% ± 0.8%	
		Actual: ≥ 1 Unplanned Visit	39.8% ± 1.0%	60.2% ± 1.0%	
		Average	65.7% ± 0.3%		
Radial SVM	Cost = 50; Gamma = 0.1	Actual: No Unplanned Visits	57.6% ± 1.4%	42.5% ± 1.4%	
		Actual: ≥ 1 Unplanned Visit	28.4% ± 0.9%	71.6% ± 0.9%	
		Average	64.6% ± 0.8%		
Single hidden layer NN	Hidden layer = 20 nodes; Iterations = 200; Decay = 0.0	Actual: No Unplanned Visits	50.7% ± 28.7%	49.3% ± 28.7%	
		Actual: ≥ 1 Unplanned Visit	31.6% ± 20.4%	68.4% ± 20.4%	
		Average	59.5% ± 7.7%		
Triple hidden layer DNN	Hidden layers = 20 nodes; Learning = 1.0; Momentum = 0.5; Iterations = 20	Actual: No Unplanned Visits	65.7% ± 14.5%	34.4% ± 14.5%	
		Actual: ≥ 1 Unplanned Visit	36.7% ± 14.6%	63.3% ± 14.6%	
		Average	64.5% ± 0.8%		
XG boost	Max depth = 20; Eta = 0.90; # rounds = 200; Gamma = 10; Min. child weight = 10; Ratio of column per tree = 1.0	Actual: No Unplanned Visits	33.9% ± 30.8%	66.1% ± 30.8%	
		Actual: ≥ 1 Unplanned Visit	16.7% ± 15.3%	83.3% ± 15.3%	
		Average	58.6% ± 7.8%		
Logistic Regression	N/A	Actual: No Unplanned Visits	60.4% ± 0.8%		39.6% ± 0.8%
		Actual: ≥ 1 Unplanned Visit	29.8% ± 0.8%		70.2% ± 0.8%
		Average	65.3% ± 0.7%		

Basic cross-validation was run for classifiers without hypermarameters (linear and quadratic discriminant analysis, logistic regression) and nested cross-validation for classifiers with hyperparameters (linear and radial SVM, single- layer NN and triple-layer DNN) to optimize hyperparameters

Cross-validation matrices show the generalization performance with respect to the actual class (rows) against the predicted class (columns), with ± standard deviation across cross-validation runs. *DNN deep nets. NN* neural nets. *SVM* support vector machines. *XG boost* extreme gradient boosting

**Table 3:** Optimal hyper-parameters across each of 5 “outer” folds in nested cross-validation

Parameter	Fold 1	Fold 2	Fold 3	Fold 4	Fold 5
*Linear SVM*					
Cost	0.1	25	25	25	25
*Radial SVM*					
Cost	25	50	50	50	50
Gamma	0.1	0.1	0.1	0.1	0.1
*Single-layer NN*					
Size of hidden layer	15	20	20	20	1
Maximum # iterations	100	200	200	200	100
Decay	0.0	0.0	0.0	0.0	0.1
*Triple-layer DNN*					
Size of 3 hidden layers	20, 20, 20	20, 20, 20	20, 20, 20	20, 20, 20	20, 20, 20
Learning rate	1	1	1	1	1
Momentum	0.5	0.5	0.5	0.5	0.5
Number of epochs	20	20	20	20	20
*XG Boost*					
Max depth	20	20	6	20	6
Eta	0.9	0.9	0.01	0.9	0.01
Nrounds	200	200	50	200	50
Gamma	10	10	0	10	0
Min. child weight	10	10	0	10	0
Ratio of columns per tree	1.0	1.0	0.1	1.0	0.1

*NN* neural nets. *DNN deep nets. SVM* support vector machines. *XG boost* extreme gradient boosting

Table 4 shows the corresponding balanced accuracy for *training* sets for the optimized classifiers shown in Table 2. Balanced accuracies are extremely similar across training and testing accuracies (different by only a fraction of a percentage point in most cases), which is one indicator of a low degree of overfitting [24].

**Table 4:** Training performance of classifiers with optimized parameters, presented as confusion matrices and balanced accuracy ± standard deviation across five-fold cross-validation runs

Classifier	Predicted: no unplanned visits	Predicted: ≥ 1 unplanned visit	
*Linear discriminant analysis*			
Actual: No Unplanned Visits	50.7% ± 1.1%	49.3% ± 1.1%	
Actual: ≥ 1 Unplanned Visit	24.7% ± 0.7%	75.3% ± 0.7%	
Average	63.0 ± 0.2%		
*Quadratic discriminant analysis*			
Actual: No Unplanned Visits	83.0% ± 0.2%	17.1% ± 0.2%	
Actual: ≥ 1 Unplanned Visit	56.2% ± 0.2%	43.8% ± 0.2%	
Average	63.4% ± 0.1%		
*Linear SVM*			
Actual: No Unplanned Visits	71.3% ± 0.8%	28.7% ± 0.8%	
Actual: ≥ 1 Unplanned Visit	39.6% ± 0.7%	60.4% ± 0.7%	
Average	65.8% ± 0.1%		
*Radial SVM*			
Actual: No Unplanned Visits	67.0% ± 1.1%	33.0% ± 1.1%	
Actual: ≥ 1 Unplanned Visit	21.4% ± 0.4%	78.6% ± 0.4%	
Average	72.8% ± 0.1%		
*Single hidden layer NN*			
Actual: No Unplanned Visits	50.8% ± 28.7%	49.2% ± 28.7%	
Actual: ≥ 1 Unplanned Visit	31.5% ± 20.2%	68.5% ± 20.2%	
Average	59.7% ± 7.9%		
*Triple hidden layer DNN*			
Actual: No Unplanned Visits	65.4% ± 15.0%	34.6% ± 15.0%	
Actual: ≥ 1 Unplanned Visit	36.5% ± 14.3%	63.5% ± 14.3%	
Average	64.4% ± 0.8%		
*XG boost*			
Actual: No Unplanned Visits	38.8% ± 35.3%	61.2% ± 35.3%	
Actual: ≥ 1 Unplanned Visit	12.9% ± 11.2%	87.2% ± 11.2%	
Average	63.0% ± 11.9%		
*Logistic regression*			
Actual: No Unplanned Visits	60.4% ± 0.2%		39.6% ± 0.2%
Actual: ≥ 1 Unplanned Visit	29.8% ± 0.2%		70.2% ± 0.2%
Average	65.3% ± 0.2%		

Basic cross-validation was run for classifiers without hypermarameters (linear and quadratic discriminant analysis, logistic regression) and nested cross-validation for classifiers with hyperparameters (linear and radial SVM, single-layer NN and triple-layer DNN) to optimize hyperparameters

Cross-validation matrices show the training performance with respect to the actual class (rows) against the predicted class (columns), with ± standard deviation across cross-validation runs. *DNN deep nets. NN* neural nets. *SVM* support vector machines. *XG boost* extreme gradient boosting

Table 5 shows the sensitivity analysis of the optimized linear SVM classifier presented in Table 2, using the optimized model to predict on each subset with normally-distributed noise added to each variable, one at a time. Both blood pressure and HDL cholesterol were found to contribute most significantly to the prediction task: adding noise to the blood pressure variable (thus disrupting its contribution to the prediction task) decreased the model’s balanced accuracy by 1.3 percentage points, and adding noise to the HDL cholesterol variable resulted in a decrease of 1.4 percentage points. The smallest change in balanced accuracy comes from LDL which when removed from the model, did not disrupt balanced accuracy at all. These analyses show that BP and HDL seem to be the most important indicators of unplanned medical visits among patients with diabetes, among the potentially modifiable variables.

**Table 5:** Sensitivity analysis showing the disruption of balanced accuracy when adding normally-distributed noise (0.3 × standard deviation) to each variable

Variable range	New balanced accuracy (%)	Change in balanced accuracy (vs. 65.8% on original sample) (%)
A1C	65.7	− 0.1
BMI	64.7	− 1.1
BP	64.5	− 1.3
HDL	64.4	− 1.4
LDL	65.8	− 0.0
Tobacco use	65.0	− 0.8

Balanced accuracy is the average of the sensitivity and specificity rates (see text), based on test sets across 25 cross-validation tests using repeated-hold-20%-out subsampling. Change in balanced accuracy is relative to the optimized classification results using the original data sample in Table 2 (65.8%)

*A1C* glycohemoglobin. *BMI* body mass index. *BP* blood pressure. *HDL* high-density lipoprotein. *LDL* low-density lipoprotein

The above findings were among many components that led to the development of a clinical intervention at the sponsoring healthcare organization. Faced with time limitations and the need to provide evidence-based recommendations to inform an intervention, the research team and the organization made the choice to forego further refinements to the predictive model and proceed with the recommendations above (i.e. targeting HDL and BP, among other factors decided by other participants in the larger process).

Moreover, practical and patient-centered considerations outweigh further gains in predictive accuracy when delivering an intervention: for example, patients often have difficulty comprehending numerical risk presented to them [25, 26]. Thus, a highly accurate model will unfortunately be ineffective if the risk is not communicated to patients in a way that they are able to understand and which would motivate behavioral changes. For this reason, the importance of improving the predictive model’s balanced accuracy became less pressing than implementation science considerations. Our research team then turned to the behavioral economics literature [27–29] to identify best practices for enrolling patients and maintaining participation in the eventual intervention.

Also important is stakeholder engagement; for an intervention to be successful, it must have the buy-in of multiple participating sectors [30, 31], including physicians, staff, and clinical operations. Physicians and healthcare staff were consulted with to understand how our model’s recommendations fit with their current standard of care and what new steps they would be willing to take in the clinic. For example, most physicians faced with patients with diabetes or pre-diabetes are already doing everything they can to improve cholesterol levels and lower BP. The operations sector was also consulted in order to identify how to streamline the intervention most easily into the existing workflows, and how to most efficiently collect essential process and outcome data while minimizing increases to provider workload. After this stakeholder engagement process, a behavioral intervention was decided on which involves shared decision-making between providers and patients to pursue one of 5 behavioral changes: weight loss; increased physical activity; nutrition counseling; smoking cessation; and medication. This intervention is currently being piloted at the sponsoring organization.

## Discussion

This study utilized machine learning to predict unplanned medical visits among patients with diabetes over a 3-year period, using readily available variables from EMRs as prediction variables. Linear-basis SVM was able to achieve slightly but significantly more accurate prediction relative to conventional logistic regression, with average balanced accuracy (average of sensitivity and specificity) of 65.7%, representing a 0.4 percentage point increase over logistic regression. Further, post-hoc analysis of the optimized prediction model revealed that HDL and BP are possibly the most important modifiable variables that predict unplanned medical visits among patients with diabetes. These recommendations from the predictive modeling were one of many components that led to the development of a clinical intervention now being piloted at the sponsoring healthcare organization.

HDL and BP may be driving unplanned medical visits among patients with diabetes due to their individual risks for unplanned medical. HDL is generally known as being the “good cholesterol” because of its atherogenesis inhibitory properties. In addition, HDL is normally anti-inflammatory; however, HDL often has a loss of function in patients with diabetes, and thus the anti-inflammatory properties are inhibited [32, 33]. The disease mechanisms in both diabetes and hypertension are similar, and have commonalities in etiology including obesity, inflammation, oxidative stress, and insulin resistance [34]. Similarly, high BP in diabetes patients is associated with increased risk of death and diabetes-related complications, which explains the finding that high BP is especially predictive of unplanned medical visits [35].

Presently, literature shows more evidence of hospitals employing predictive analytics related to reducing emergency care utilization. Though not exactly comparable to the current study’s aim of predicting unplanned medical visits among patients with diabetes, similar applications such as the HOSPITAL and LACE screening tools predict emergency room readmission risk. The HOSPITAL score uses seven clinical predictors to help identify patients at high risk of hospital readmissions within 30 days of discharge. This score has been validated and shown to have superior discriminative ability over other prediction tools [36]. Similarly, the LACE index uses only four variables to predict death or 30-day readmission after hospital discharge of 66.3% and a correct rejection rate of 53.3% [37]. While this tool has also been validated, LACE has been shown to only have moderate discriminative ability [36]. This demonstrates the utility of such predictive models, including the current study’s model for predicting unplanned medical visits among patients with diabetes.

Therefore, the higher prediction balanced accuracy in the current model demonstrates the utility of machine learning approaches for prediction of medical risks. Though the improvement in balanced accuracy may be considered small (~ 0.4 percentage points), this difference was statistically significant and could have substantial implications at a large scale. For example, back-of-the envelope calculations show that, under the assumption that these visits can be anticipated and prevented with perfect accuracy, an improvement of 0.4% for a population of 1 million patients with diabetes, given an unplanned visit rate of 57.2% (based on this sample), translates into approximately 2300 people and 7500 visits that could be avoided.

The higher accuracy is likely attributable to the increased predictive information contained in *patterns* of variables, over and above each variable’s statistically independent association with the outcome [11, 38]. Though this pattern-based information is difficult to extract in “black-box” models (e.g. SVM), we present a form of sensitivity analysis that estimates each variable’s total contribution to the model (accumulated across its statistically independent main effect and all interactions with other predictor variables) and thus can quantify each variable’s “diagnostic information.”

Identifying the most salient predictors is an important step towards moving this predictive algorithm into concrete implementation in clinical settings. That is, the trained predictive model can be used for hypothesis generation (i.e. that risky HDL and BP values lead to unplanned medical visits). Since the predictive model itself cannot test or establish causality, further longitudinal research in clinical settings is needed to test these hypotheses; nevertheless, this hypothesis generation is an essential step in that it reduces the number of likely hypotheses that must be tested in clinical settings, leading to a more efficient use of resources. Following the hypothesis validation stage, an evidence-based intervention then can be designed and implemented which flags high-risk patients for an appropriate protocol (e.g. more aggressive targeting of BP and HDL through clinical or behavioral measures).

The above recommendations were one component of many in the development of a clinical intervention for at-risk patients, along with physician engagement, interfacing with clinical operations, and utilizing behavioral economics to maximize patient engagement with the intervention. Practical limitations in this project are common to many other projects which seek to translate research into clinical practice; most notably in the current study, the trade-off between focusing efforts on improving model accuracy versus focusing efforts on implementation science to ultimately maximize patient engagement. Thus, this study demonstrates the value of this study’s approach not only in improved prediction of costly unplanned medical visits, but also in moving towards clinical implementation.

### Limitations

This study has several limitations. First, causality cannot be established using observational data; however, the current procedure of performing a sensitivity analysis on modifiable predictor variables produces a more refined set of causal hypotheses that can be pursued in follow-up research. A related limitation is that factors that may be relevant for prediction may not be pertinent for treatment (e.g. age which is not modifiable). Additionally, results may not be generalizable to other populations outside the North Dakota, South Dakota, and Minnesota, and further validation is needed in other independent samples.

Other methodological limitations exist which, if rectified, could improve models’ balanced accuracy and predictive power. Specifically, the types of classifiers used here are not comprehensive, and other methods such as random forests could offer improvements in predictive accuracy. Within the existing classifiers, it is also possible that optimizing across much wider parameter ranges could lead to higher balanced accuracy. When taking these methodological limitations into account, the marginal gains of model accuracy must be weighed against practical considerations if being used in clinical settings.

Limitations of EMR data are numerous, and this is unfortunately common when using EMR data for research. The data available had limited variables, such as imprecise measures of smoking status; and the unavailability of other variables including socio-economic and behavioral determinants of health, disease duration and severity, and depression in the current EMR system, are likely to negatively impact the prediction accuracy. However, basing our prediction model on standard EMR fields increases its utility within this healthcare system, as well as its potential generalizability of these methods to other healthcare systems. The current data were also limited with respect to the granularity of time-varying variables (e.g. BMI, BP): namely, in the dataset provided by the sponsoring healthcare organization, only the last time point was provided. This is problematic because for true prediction, the predictors must precede the outcome in time. However, many of these factors are fairly stable over time, lessening the impact of this limitation. Further, this and other data limitations are common in health services research, and this study provides a practical example of how clinical implications can be generated from a predictive model in spite of realistic data limitations. Another limitation is that the outcome variable of unplanned medical visits does not consider the *cause* of the visit; thus, visits may or may not be related to diabetes. Though there is some literature on identifying preventable emergency visits, this is a difficult process, and to our knowledge no method exists for identifying diabetes-attributable visits. Therefore, we analyze all unplanned visits together, which represents a realistic situation when analyzing EMR data. These data limitations are common for administrative health records; thus, the current study is practical in the sense that it is representative of working with real-world data limitations. However, more rigorous data with fewer of the above limitations can improve predictive modeling. Thus, improvements to data collection and querying processes and capabilities should be a priority for the use of administrative health records in research.

### Strengths

The use of EMR data from a large healthcare system in the US allows for the capture of large proportion of the population, and a large sample size. This study also utilizes innovative machine learning methods with cross-validation, which leads to improved prediction accuracy and generalizability of results. Finally, the current study demonstrates a relatively novel procedure for moving a machine-learning model from pure prediction towards making clinical improvements to care management.

## Conclusions

This study shows improved prediction of unplanned medical visits among patients with diabetes by utilizing machine learning methods, relative to conventional prediction models. A post-hoc sensitivity analysis identified low HDL and high BP as the strongest predictors of unplanned medical visits among this patient population, warranting future research in clinical settings on whether these are causal relationships. Future research is underway based on this predictive model on a behavioral health intervention aimed at improving diabetes management in clinical settings. Improvements are needed to standard data collection and querying procedures for administrative health records in order to overcome important data limitations that limit current predictive modeling.

## Data Availability

The datasets used during the current study can be applied for through the Sanford Data Collaborative: https://research.sanfordhealth.org/services/sanford-data-collaborative. The code used in this study is freely available at: https://github.com/ArielleSelya/Diabetes-Predictive-Model.

## Abbreviations

EMR: Electronic medical records
BMI: Body mass index
BP: Blood pressure
LDL: Low-density lipoprotein
HDL: High-density lipoprotein
A1C: Glycohemoglobin
MN: Minnesota
ND: North Dakota
SD: South Dakota
SVM: Support vector machine
NN: Neural network
XG boost: Extreme gradient boosting
